# Optimization of the sterilizing doses and overflooding ratios for the South American fruit fly

**DOI:** 10.1371/journal.pone.0201026

**Published:** 2018-07-20

**Authors:** Thiago Mastrangelo, Adalecio Kovaleski, Victor Botteon, Wanessa Scopel, Maria de Lourdes Zamboni Costa

**Affiliations:** 1 Centro de Energia Nuclear na Agricultura (CENA), Universidade de São Paulo, Piracicaba, São Paulo, Brazil; 2 Empresa Brasileira de Pesquisa Agropecuária (EMBRAPA), Estação Experimental de Fruticultura de Clima Temperado, Vacaria, Rio Grande do Sul, Brazil; 3 Escola Superior de Agricultura ‘Luiz de Queiroz’ (ESALQ), Universidade de São Paulo, Piracicaba, São Paulo, Brazil; University of Thessaly School of Agricultural Sciences, GREECE

## Abstract

The Sterile Insect Technique (SIT) is an autocidal control method that relies on inundative releases of sterilized mass-reared insects. This technology has been used in several area-wide programmes for the suppression/eradication of fruit fly populations. Choosing the optimum sterilizing dose and the sterile release density is an essential step of the SIT. Considering unsolved issues related to the application of this technique against *Anastrepha fraterculus* (Wiedemann), this study aimed to define accurately the central target dose for both sexes of this species and to verify the induction of sterility in fertile flies at different sterile:fertile ratios. The results from the regression analyses proved that the sterilization process for the *A*. *fraterculus* Brazilian-1 morphotype (the most common in southern Brazil and Argentina) could consist of irradiating pupae 72 h before adult emergence at 40 Gy, with no detrimental effects to standard quality control parameters. The ovarian development in irradiated females was characterized, demonstrating that doses equal to or higher than 25 Gy cause complete and irreversible ovarian atrophy. The laboratory and field cage tests showed that the sterility induction increased with the proportion of sterile flies, and a sterile:fertile ratio of 50:1 should be appropriate in SIT field trials. The sterile females apparently did not distract the sterile males, despite of the slightly higher reductions in pupal yield for all ratios in their absence. The data generated in this study have a great practical value and will help decision-makers in planning field trials to evaluate the efficacy of the SIT against *A*. *fraterculus* populations.

## Introduction

The Sterile Insect Technique (SIT) is an autocidal control technology that relies on inundative releases of sterilized mass-reared insects [1]. This technology has been integrated in many area-wide integrated pest management (AW-IPM) programmes against a number of fruit fly species. For the Mediterranean fruit fly, *Ceratitis capitata* (Wiedemann), several programmes have successfully applied the SIT to promote its eradication/ containment in Argentina, Chile, Peru, Mexico and Guatemala, and for preventing its establishment in California and Florida [2]. In Mexico, millions of sterile flies are produced per week at the MOSCAFRUT facility for the control of economically important *Anastrepha* species [3].

With the eradication of the codling moth, *Cydia pomonella* (Linnaeus), achieved in 2014 after a 17-year campaign in Brazil [4], researchers and growers decided to create the MOSCASUL center at Vacaria, Rio Grande do Sul, Brazil. The main goal of this center is to suppress the wild populations of the South American Fruit Fly, *Anastrepha fraterculus* (Wiedemann), from temperate fruit production areas of southern Brazil through the application of sterile insects and parasitoids [5]. Before scaling up the rearing colonies and the release of sterile *A*. *fraterculus* flies in the fields, many technical issues must still be revised or optimized.

Ionizing radiations, such as gamma or X-rays, have been used to induce sterility in mass-reared insects worldwide [6]. The selection of the sterilizing dose is extremely important to SIT programmes. Research is essential to clarify the relationship between dose and the level of sterility in the irradiated flies. Dose-response curves for sterility can be developed by irradiating flies with increasing doses, mating them separately with nonirradiated flies, and calculating egg hatch [7]. In general, the sterilization curves approximate to linearity at low doses, but at higher doses they approach 100% sterility asymptotically and to eliminate a residual egg hatch of 1% (or less) from fertile females mated to irradiated males usually requires much higher doses [6]. As the radiation doses increase, more and more they negatively affect the vigour and/or competitiveness of the insects [8]. At too-low doses, the flies are not sufficiently sterile and cannot be released in the field, as the accidental release of under-dosed females could compromise the effectiveness of the technique and even damage crops. Based on the results from radiation studies, the managers of SIT programmes can specify the optimum dose that balances sterility with insect vigour.

The sterilization of *A*. *fraterculus* from Argentinean colonies had been addressed by two studies with different methodologies and analyses [9]. Allinghi et al. [10] verified the effects of gamma radiation (only at 50, 70 and 90 Gy) over pupal ages (96, 72, 48 and 24 h before adult emergence) and, in a 2^nd^ experiment, they estimated male and female sterility after irradiating pupae 48 h before emergence with other short range of doses (20, 40 and 60 Gy). The egg hatch data from the 1^st^ experiment were analyzed by a homogeneity chi-square test, while egg viability from the 2^nd^ experiment was evaluated with a non-parametric Kruskall-Wallis analysis. Neither were regression analyses performed nor dosimetry and quality control parameters reported in the study. Their results indicated that pupal age at irradiation did not affect male sterility and the authors concluded that full sterility in both sexes could be achieved only with a dose of 70 Gy.

Mastrangelo et al. [11] compared dose-response curves for both sexes of *A*. *fraterculus*, irradiated as pupae 48–24 h before emergence with four doses (10, 20, 35 and 70 Gy) of gamma or X-rays. Dosimetry of irradiations was performed according to the Gafchromic^®^ dosimetry system [12]. The standard quality control parameters [13] and mating indices were not affected by the two types of radiation. The estimated SD_99_ values (*i*.*e*. the estimated dose that induces 99% sterility) for males were 36–37 Gy and 57–58 Gy for females. This difference in the sterilizing doses between sexes was unusual, because female insects are generally more radiosensitive than males [6]. According to the authors, the fewer doses applied to fit the regression curves for irradiated females might have increased the errors at high doses, which led to the overestimation of SD_90_ and SD_99_values.

As genetic sexing strains (GSS) for *A*. *fraterculus* are not yet available to be field released, the MOSCASUL center will depend initially on bisexual strains to conduct suppression pilot trials. A minimum dose that all pupae must receive has to be specified to ensure sufficient sterility in both male and female flies, without reducing the competitiveness of the males. Preliminary release-recapture trials performed at Vacaria in 2017 with flies irradiated with 70–80 Gy revealed that they had a very poor dispersal ability in forested areas surrounding apple orchards (Mastrangelo, unpublished data). Therefore, in view of the different sterilizing doses obtained by the previous studies, an optimum dose should be defined more accurately for *A*. *fraterculus* males and females in order to be officially recommended for AW-IPM projects against this pest.

An accurate estimate of the optimum ratio of sterile-wild males (*i*.*e*. overflooding ratio, hereafter OFR) is another technical issue essential for any SIT programme. When sterile males are released, they must find and mate with the wild females, making their offspring not viable. In control operations, the induction of sterility into the wild population becomes more efficient when the sterile males sufficiently outnumber the native flies after the constant releases, ensuring progressive population decline over the generations [14]. Several studies have provided estimates of the OFRs needed to control *C*. *capitata* and *Bactrocera* spp. [15], but only Flores et al. [16, 17] estimated appropriate OFRs for *Anastrepha* species (*i*.*e*. *Anastrepha ludens* (Loew) and *Anastrepha obliqua* (McQuart). So far, no study relating the induction of sterility and OFRs has been conducted for *A*. *fraterculus*.

Considering these unsolved problems related to the SIT against *A*. *fraterculus* and the urgent need of more technical data for the field trials of the MOSCASUL project, the goals of this study were: 1) to define accurately the central target dose and the best age of pupae at the time of irradiation for both sexes of a local *A*. *fraterculus* Brazilian-1 morphotype, also characterizing the morphology of the ovaries after treatment at low doses; and 2) to verify the induction of sterility in fertile flies at different OFRs in laboratory cages and under field cage conditions.

## Materials and methods

### Insects and irradiation procedures

The experiments took place at the *Food Irradiation & Radioentomology Laboratory* from the Center for Nuclear Energy in Agriculture (CENA), Piracicaba, Brazil. The *A*. *fraterculus* pupae used for the experiments were obtained from a colony maintained following the procedures described by Walder et al. [18]. This bisexual strain of the Brazilian-1 morphotype (*i*.*e*. the most common morphotype of the *A*. *fraterculus* complex in Argentina and southern Brazil) [19] was originally established with pupae recovered from native infested hosts at Vacaria and maintained in the laboratory for 18 generations. No permits were required for the described study, which complied with all relevant regulations.

The pupae for the experiments were irradiated with X-rays generated by an RS-2400V irradiator (RadSource Technologies Inc., Buford, GA), operated at 160 keV and 45 mA giving a rate of 13.1±0.4 Gy/min at the irradiation position. The absorbed dose distribution in the canisters and dose rates were determined following the procedures described by Mehta & Parker [20]. All irradiations were carried out separately for the different bioassays and performed under normal atmospheric conditions (21% oxygen). A small plastic container with the pupae was positioned in the center of the canisters (7.6 cm diameter x 20.3 cm long) that were suspended by a carousel that rotates around the X-ray source. For each exposure, dosimetry was performed following the Gafchromic^®^ dosimetry system [12].

### Optimization of the sterilizing doses for *A*. *fraterculus*

Pupae with different ages (72, 48 and 24 h before adult emergence) were treated with six doses of X-rays: 0 (control), 5, 10, 15, 25, 35 and 45 Gy. Two days after emergence, adults were sorted by sex. For each age and dose, fertility was assessed by exposing 25 nonirradiated females or males to 25 irradiated individuals of the opposite sex for a week in 2 L plastic cylinder flasks. Water and food were offered *ad libitum*, being the later a mixture of hydrolysate yeast (*Bionis*^®^ YE MF, *Biorigin*, Lençóis Paulista, SP), sugar and wheat germ (1:3:1). Females laid eggs through netting on the top of the cage into a water-filled dish whose base was a red cloth covered with a thin layer of silicone rubber. The water from this oviposition device was changed every 24 h and the eggs were collected six times for each treatment. The eggs from each cage were counted on a moist filter paper and placed over a moistened dark sponge in petri dishes. The hatchability of eggs was assessed after 7 days (25 ± 1 °C and 70% RH). The ovipositing cages were distributed in a randomized design. Each treatment was replicated four times, using pupal cohorts from different generations (one replication per cohort).

To assess adult emergence and sex ratio from irradiated pupae, 100 pupae for each age and dose were placed in petri dishes, with three replicates per treatment. The percentage of adult emergence and sex ratio were calculated four days later. To assess the percentage of fliers, 50 pupae from each treatment were positioned in the bottom of black Plexiglas tubes (8.9 cm diameter x 10 cm high) whose walls were coated with unscented talcum powder [13]. After emerged flies had flown from the tubes, the remaining flies and unemerged pupae were counted. Three replicates per treatment were distributed in a randomized design.

To verify the effects of radiation on the competitiveness of males and females, pupae 72–48 h before adult emergence were irradiated with the selected SD_99_ (*i*.*e*. 40 Gy) and the emerging flies were kept in separate cages. When the flies were 10-d-old, 25 nonirradiated and irradiated couples, marked with water-based paint dots [13] from the same strain were released in circular screened field cages (3 m diameter x 2 m high), each containing a potted apple tree (*Malus domestica* Borkh cv.‘Gala’) of 1.5 m in height and a canopy of 0.5 m in diameter. Females were released 30 min. after the release of males. The type and number of matings were recorded for 4 h (7:30 to 11:30 a.m.). The index of sexual isolation (ISI), male and female relative performance indices (MRPI and FRPI), and the relative isolation index (RII) were estimated according to the manual of FAO/IAEA/USDA [13]. There were 12 replicates (one replication per cohort) for each index.

### Characterization of the ovaries from irradiated flies

The batches of pupae 48 h before emergence destined for the tests were irradiated with 0 (control), 15, 25, 35, and 45 Gy. Twelve hours after initial emergence, adults were sorted by sex. For each radiation dose, 1-d-old nonirradiated males were placed with irradiated females of the same age in cages made of a 2 L plastic cylinder flask. Control cages with nonirradiated flies were also set up. In each cage, the adults were fed *ad libitum* with water and adult diet, as above. These cages were distributed in a randomized design with 4 replicates for each treatment, with 25 couples per cage.

Adult females were dissected in 0.85% sodium chloride (NaCl) solution [21] at 1, 7, and 15 days of age. After removal of the reproductive system, the ovaries were transferred to a clean microscope slide and examined as whole mounts under a Leica MDG41^®^ stereomicroscope (Leica Microsystems, Heerbrugg, Switzerland). Images of the freshly dissected organs were made with a Leica Digital DFC450^®^ camera on the stereomicroscope for further characterization. The length (mm), width (mm) and area (mm^2^) of each ovary on the three different days were measured from the images.

### Sterility induction at different sterile: Fertile ratios in laboratory cages

For the evaluation of sterility induction at different OFRs in controlled environmental conditions, pupae were irradiated 72 h before emergence with 40 Gy (this age and dose were chosen based on the results from the previous experiments). Two days after emergence, the adults were sorted and fertility was assessed exposing the irradiated males to nonirradiated males and females in cages with the ratios presented in Table 1.

**Table 1:** Ratios of sterile and fertile *Anastrepha fraterculus* flies by laboratory cage.

Ratio	Sterile	Fertile		Total number of flies
	♂	♂	♀	
1:1:1	26	26	26	78
5:1:1	120	24	24	168
10:1:1	330	33	33	396
30:1:1	930	31	31	992
50:1:1	10,200	204	204	10,608
100:1:1	20,200	202	202	20,604
(control) 0:1:1	0	25	25	50
(sterile control) 1:0:1	25	0	25	50

The flies for the ratio of 1:1 and control groups were placed in the 2 L plastic cylinder cages, 45x45x45 cm screened cages were used for the ratios of 5:1, 10:1 and 30:1, and colony cages (75x30x154 cm) [18] were used for the ratios 50:1 and 100:1. The flies were fed *ad libitum* as previously described and the same oviposition devices made of water-filled dishes were used when the flies reached the age of 10–11 days. Eggs were collected daily for six consecutive days. Egg hatch was evaluated after 7 days. Four replicates were performed per cage and different cohorts were used for each replicate.

### Sterility induction at different sterile: Fertile ratios under field cage conditions

The treatments varied in their sterile:fertile ratios and in the presence or absence of sterile females. The number of released flies in each field cage is shown in Table 2.

**Table 2:** Ratios of sterile and fertile *Anastrepha fraterculus* flies by field cage.

Field Cage Test	Ratio	Sterile		Fertile		Total number of flies
		♂	♀	♂	♀	
**Sterile male and female release**	(control) 0:0:1:1	0	0	50	50	100
	1:1:1:1	50	50	50	50	200
	10:10:1:1	490	490	49	49	1,078
	42:42:1:1	2,100	2,100	50	50	4,300
	50:50:1:1	2,500	2,500	50	50	5,100
	100:100:1:1	5,100	5,100	51	51	10,302
**Sterile male only release**	(control) 0:0:1:1	0	0	50	50	100
	1:0:1:1	50	0	50	50	150
	10:0:1:1	490	0	49	49	588
	42:0:1:1	2,100	0	50	50	2,200
	50:0:1:1	2,500	0	50	50	2,600
	100:0:1:1	5,000	0	50	50	5,100

Pupae 72 h before emergence were X-rayed with 40 Gy to render the flies sterile. Fertile flies from the laboratory bisexual strain were used in lieu of wild flies because sufficient numbers of wild flies were not available during the tests. Batches of irradiated and nonirradiated pupae were placed separately in 50x50x50 cm acrylic cages and kept at 25 °C in the laboratory. At emergence, the flies were sorted by sex and the exact numbers required by the tests were placed in 45x45x45 cm screened cages. Water was provided by vials with cotton wicks and the adult diet was the same from the previous tests. Before the tests, the sexes were kept isolated in separate cages to reach sexual maturation and to avoid any contact of pheromone under laboratory conditions (25±1 °C, 65% RH and a photoperiod of 12:12 [L:D] h).

The tests with the presence or not of sterile females were conducted independently due to the limited number of field cages. Sexually mature 10-d-old flies were released in the same circular screened field cages described previously. The field cages were installed under the shade of trees from an Atlantic forest fragment in the ‘Luiz de Queiroz’ campus, Piracicaba (latitude 22°42’ S and longitude 47°38’W). According to the number of flies in Table 2, males were released first into each of the cages, followed by females after an interval of 20 min. Dead flies and those incapable of flying or noticeably damaged in any way at the time of release were replaced. Water and adult diet were supplied in each cage at the base of the potted tree. Two days after release, one papaya fruit (*Carica papaya* L. cv.‘Golden’) at maturity stage 3 [22, 23] with average weight of 493.5±19.2 g was hung on each caged tree and replaced twice after three consecutive days of exposure. These fruits were then transported to the laboratory, placed on screens above vermiculite in individual boxes and maintained for 15–17 days at 25–26 °C. The vermiculite was sifted every 2–3 d for pupae. Eight replicates were carried out per treatment in time using different pupal cohorts. Pupae recovered from the fruits were counted and stored in petri dishes at the laboratory (25±1 °C and 65% RH). As the percentage of adult emergence of the pupae recovered, irrespective of the treatment, was ca. 98–100%, only data for recorded number of pupae were included in the statistical analyses. The sunshine records, temperature and relative humidity during the tests were favorable for fly requirements.

### Data analysis

The egg hatch data from the tests for optimization of the sterilizing dose and sterility induction at different OFRs in laboratory cages were corrected for the respective control values [24] and Probit transformed for the performance of linear regression analyses against the log of the radiation dose or the sterile:fertile male ratio, respectively [25]. Based on the dose-response curves obtained, the estimated doses and ratios that induce 50%, 90% and 99% sterility (*i*.*e*. SD_50_, SD_90_ and SD_99_) were calculated with their 95% confidence intervals. For the percentages of adult emergence, fliers and sex ratio, linear regression analysis was applied. For the length (mm), width (mm) and area (mm^2^) of the ovaries, the one-way analysis of variance *F*-test was applied at the 5% significance level (ANOVA) and, when significant differences were found, Tukey’s honestly significance difference (HSD) test (α = 5%) was used to compare the means.

After the infestation of papaya fruits in the field cage tests, the percentage of reduction in pupal production was estimated with the formula: 100%-(1- number of pupae recovered in the treatment/ number of pupae recovered in control) [26]. The relationship between the number of pupae recovered, reduction in pupal production and number of pupae/kg of fruit were adjusted to regressions with logarithmic/exponential functional forms. The equality of slopes from the regression equations in the presence or absence of sterile females were compared by a slope comparison test (α = 5%) [25]. The Bartlett and Shapiro-Wilk tests [27, 28] were performed to verify the homoscedasticity assumptions and the normality of the errors, respectively. These tests and ANOVAs were performed by the statistical program SAS 9.4 [29].

## Results

### Effects of pupal age and doses on *A*. *fraterculus* fertility and quality control parameters

As the 95% confidence intervals of all the Gafchromic dosimeters included the respective target dose, the target doses values are used throughout. The linear regression equations of the Probit transform of sterility against the logarithm of dose for the different pupal ages and the estimated SD_50_, SD_90_ and SD_99_ values are presented in Table 3 and S1, S2 and S3 Figs.

**Table 3:** Linear regression equations of Probit sterility on log dose and estimated doses at selected sterility levels for males and females of *Anastrepha fraterculus* whose pupae were irradiated at three different times (72, 48 and 24 h before adult emergence).

Pupal age (hours before adult emergence)	Sex	Probit linear regression analysis	SD_50_^†^	SD_90_	SD_99_
**72 h**	♂	y = 3.1x + 2.4; r^2^ = 0.83; *F*_1,14_ = 67.9; *P* < 10^−3^	6.8 (4.9; 9.5) ^‡^	17.7 (14.9; 21.1)	38.5 (30.4; 48.6)
	♀	y = 4.7x +0.94; r^2^ = 0.60; *F*_1,8_ = 10.7; *P* = 0.011	7.3 (4.8; 11.3)	13.8 (8.9; 21.3)	23.02 (11.5; 46.1)
**48 h**	♂	y = 3.5x + 1.8; r^2^ = 0.84; *F*_1,14_ = 75.6; *P* < 10^−3^	8.2 (6.2; 10.8)	18.9 (16.1; 22.2)	37.3 (30.1; 46.3)
	♀	y = 4.9x +0.68; r^2^ = 0.78; *F*_1,8_ = 12.9; *P* = 0.0071	7.5 (5.04; 11.0)	13.5 (9.2; 19.9)	21.9 (11.9; 40.4)
**24 h**	♂	y = 3.1x + 2.4; r^2^ = 0.81; *F*_1,14_ = 58.9; *P* < 10^−3^	6.9 (4.9; 9.9)	17.9 (14.9; 21.5)	38.6 (30.1; 49.8)
	♀	y = 4.5x + 1.3; r^2^ = 0.69; *F*_1,8_ = 7.3; *P* = 0.003	6.8 (3.8; 11.9)	13.0 (7.9; 21.5)	22.2 (9.8; 50.1)

^†^ SD = dose (Gy) that induces 50, 90 or 99% sterility.

^‡^ 95% confidence level.

The overall control fertility in the *A*. *fraterculus* laboratory strain tested was 88.8±4.2% (mean±SE), resulting in small corrections to the sterility data using the Abbot’s formula. The observed fecundity in both control groups and cages where fertile females were crossed with irradiated males was 24.3±8.6 eggs/d/female with no noticeable variations. The SD_50_, SD_90_ and SD_99_ values for males and females did not differ significantly within each sex at the same levels of sterility as the confidence intervals overlapped (Table 3). These results indicated that the three pupal ages did not affect the sterility induced in both males and females at the time of irradiation.

Considering the crosses between irradiated males and nonirradiated females, the raw sterility values induced by the doses of 5, 10, 15, 25, 35 and 45 Gy were 38.3±2.7%, 75.3±0.6%, 87.8±1.6%, 92.6±0.6%; 96.4±0.2% and 99.8±0.2%, respectively. Based on the log-probit regressions obtained, estimated doses between 37.3 Gy and 38.6 Gy could induce 99% sterility in males.

When irradiated *A*. *fraterculus* females were mated with fertile males, there were significant reductions in fertility (≈ 50%) at estimated doses as low as 6.8–8.2 Gy (Table 3) compared to the control. Only 1% of eggs hatched at the estimated doses of 21.9–23.02 Gy. No eggs were laid after irradiation with 25 Gy during the six days of egg collection.

The mean values from the quality control parameters evaluated for the irradiated pupae are summarized in Table 4. Within the pupal ages, the fitted regression line slopes did not differ from zero. Therefore, the adult emergence, fliers and sex ratio were not affected by radiation, with overall means of 84.9±0.9%, 89.2±0.93% and 0.52±0.014, respectively.

**Table 4:** Means (±SE) of emergence of flies, fliers and sex ratio from pupae of *Anastrepha fraterculus* irradiated with different doses at different ages (72, 48 and 24 h before adult emergence).

Dose (Gy)	Emergence (%)			Fliers (%)			Sex ratio (♀/♂+♀)		
	72 h	48 h	24 h	72 h	48 h	24 h	72 h	48 h	24 h
**Control**	86.7±4.4	99±1.0	88.0±4.1	80.3±4.3	99.9±0.1	97.6±2.4	0.48±0.03	0.57±0.08	0.56±0.06
**5**	86 ± 4.2	79.3±0.7	82 ±2.0	85.1±7.9	91.6±5.1	88.8±4.7	0.45±0.12	0.44±0.06	0.47±0.08
**10**	88 ± 1.2	90.0±3.1	86.7±4.4	88.7±2.6	89.6±0.9	91.1±4.5	0.52±0.01	0.69±0.06	0.48±0.07
**15**	89.3±2.4	83.3±2.9	85.3±2.9	80.8±4.1	85.7±2.0	93.1±3.3	0.46±0.03	0.36±0.01	0.61±0.03
**25**	86 ± 3.1	85.3±4.7	82.7±2.4	90.8±1.0	91.6±1.7	96 ±1.5	0.47±0.04	0.51±0.08	0.62±0.06
**35**	86.7±4.4	75.3±7.1	80.0±0.1	89.6±4.0	84.5±4.0	85 ± 3.8	0.57±0.03	0.57±0.03	0.50±0.03
**45**	88.7±1.8	75.2±2.7	84.7±3.5	87.1±5.3	85.8±2.2	96.6±3.4	0.58±0.07	0.58±0.1	0.51±0.06
**Significance test for the linear regression**^†^	*F*_1,20_ = 0.08^*ns*^ *P* = 0.78	*F*_1,19_ = 9.1^*ns*^ *P* = 0.07	*F*_1,20_ = 0.95^*ns*^ *P* = 0.34	*F*_1,20_ = 1.71^*ns*^ *P* = 0.21	*F*_1,19_ = 6.5^*ns*^ *P* = 0.21	*F*_1,20_ = 0.001^*ns*^ *P* = 0.97	*F*_1,20_ = 3.2^*ns*^ *P* = 0.09	*F*_1,20_ = 0.19^*ns*^ *P* = 0.67	*F*_1,20_ = 0.001^*ns*^ *P* = 0.98

^†^ Analyses of variance with *F*-test indicates if a significant linear regression can be fitted to the data or not (*P* > 0.05; *ns*, not significant).

The results from the mating tests conducted under field cage conditions demonstrated that a dose of 40 Gy did not severely affect the competitiveness of the flies. The mean ISI value of -0.10±0.16 indicated that the irradiated flies remained compatible with the nonirradiated flies (S4 Fig). Irradiated males were as competitive in obtaining mates as nonirradiated males (MRPI = -0.15±0.065). The sterile females demonstrated equal mating propensity, since the mean FRPI value of -0.29±0.17 was close to zero (S5 Fig). The mean RII was 1.39±1.05, also indicating random mating (S6 Fig).

### Ovarian development in irradiated and nonirradiated females

**Fig 1:**
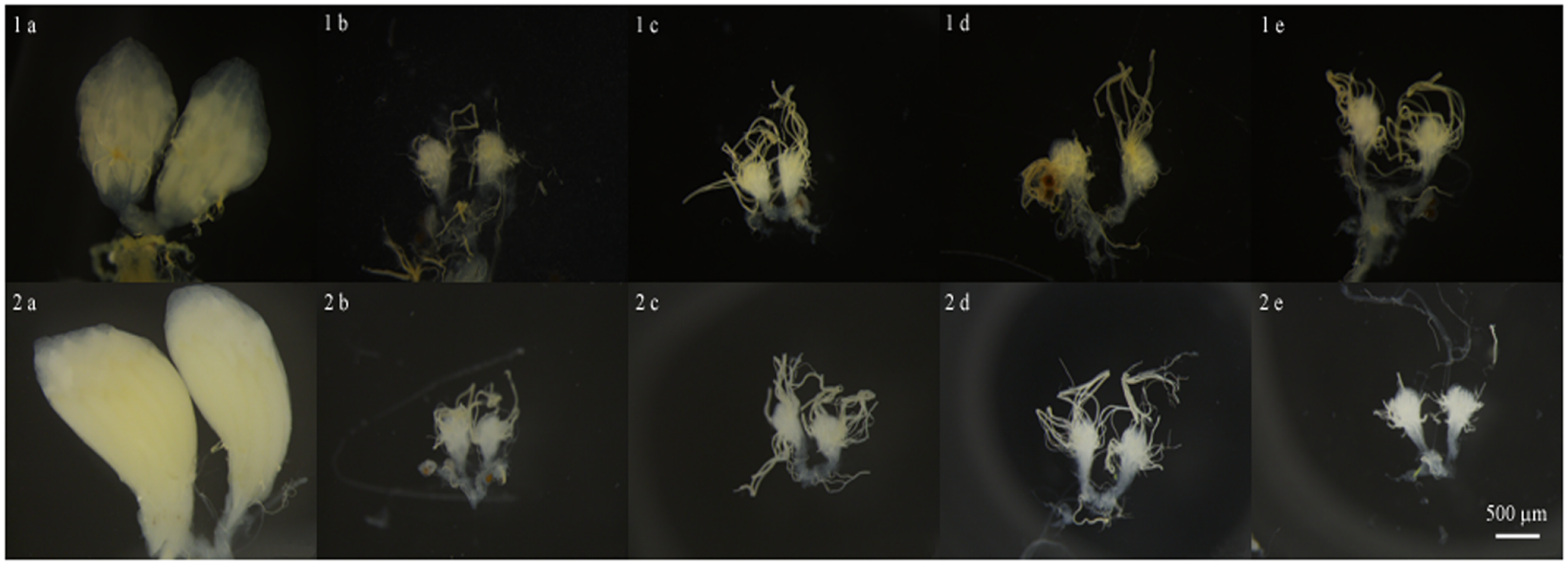
Ovarian development of *Anastrepha fraterculus* irradiated as pupae 48 hours before adult emergence with different doses (indicated by letters: a = control; b = 15 Gy; c = 25 Gy; d = 35 Gy; e = 45 Gy) and observed at different times (indicated by numbers: 1 = 7-day-old flies; 2 = 15-day-old flies).

The effects of radiation on the ovarian development of *A*. *fraterculus* are shown in Table 5 and Fig 1. On the 1^st^ day after the emergence of the adults, no morphological differences were observed among irradiated and nonirradiated ovaries. The overall mean values for the length, width and area of the ovaries from the treatments were 0.4±0.03 mm (*F*_3, 20_ = 0.3, *P* = 0.9), 0.32±0.02 mm (*F*_3, 19_ = 2.5, *P* = 0.1) and 0.1±0.007 mm^2^ (*F*_3, 20_ = 3.02, *P* = 0.06).

**Table 5:** Measurements (means ± SE) of the ovaries from fertile and irradiated females of *Anastrepha fraterculus* at two different times (7 and 15 days after the emergence of the adults).

Measurement	Age of the fly (days old)	Treatment					
		Control	15 Gy	25 Gy	35 Gy	45 Gy	ANOVA
**Length (mm)**	7	1.49 ± 0.08 a^†^	0.47 ±0.01 b	0.45 ± 0.02 b	0.44 ± 0.02 b	0.44 ± 0.01 b	*F*_4,24_ = 166.8; *P*<10^−3^
	15	2.64 ± 0.13 a	0.5 ± 0.02 b	0.51 ± 0.01 b	0.41 ± 0.03 b	0.48 ± 0.01 b	*F*_4,27_ = 247.5; *P*<10^−3^
**Width (mm)**	7	0.71 ± 0.1 a	0.27 ±0.01 b	0.28 ± 0.01 b	0.32 ± 0.01 b	0.29 ± 0.01 b	*F*_4,24_ = 15.9; *P*<10^−3^
	15	0.99 ± 0.06 a	0.39 ±0.06 b	0.28 ±0.01 b	0.25 ± 0.01 b	0.29 ± 0.02 b	*F*_4,27_ = 63.1; *P*<10^−3^
**Area (mm**^**2**^**)**	7	0.92 ± 0.05 a	0.09 ± 0.002 b	0.093 ± 0.005 b	0.106 ± 0.005 b	0.097 ± 0.01 b	*F*_4,24_ = 221.9; *P*<10^−3^
	15	1.99 ± 0.09 a	0.15 ±0.02 b	0.11±0.004 b	0.10 ± 0.01 b	0.12 ± 0.002 b	*F*_4,27_ = 321.4; *P*<10^−3^

^†^ Means (± SE) within rows followed by the same letter do not differ significantly at the 5% level (Tukey’s test).

On the 7^th^ day, however, the nonirradiated (control) ovaries were larger than the irradiated ones, and abnormally developed ovaries with few ovarioles could already be observed in females exposed to 15 Gy. The doses of 15 Gy and higher caused damage to the ovaries, making them smaller. The length, width and area of the ovaries from the flies irradiated with 15 Gy or higher did not differ significantly among them, but they did differ significantly from the means of the control group (Table 5). On the 7^th^ day, the irradiated ovaries presented mean area values 9–10 fold smaller than the values of the nonirradiated ovaries. Doses of 25 Gy or higher induced complete atrophy of the germinal cell structures and, on the 15^th^ day of observation, no female showed ovaries with signs of regeneration (Fig 1).

### Sterility induction in laboratory cages

**Fig 2:**
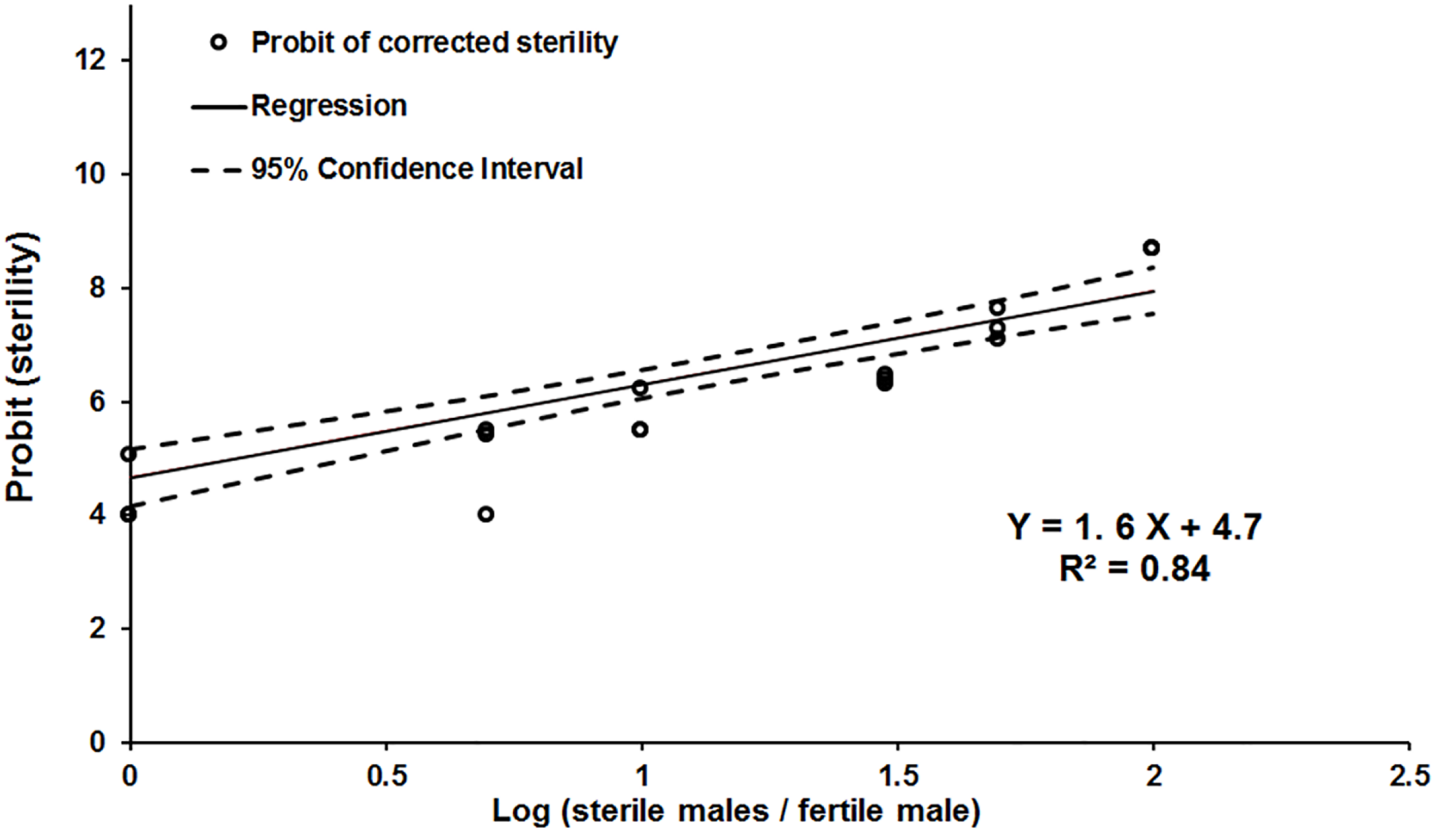
Linear regression of Probit transformed sterility on log of sterile: Fertile male ratio under laboratory conditions, without the presence of sterile females.

The percentages of egg hatch (means±SE) and the total number of eggs scored from the control cages and the ratios of 1:1, 5:1, 10:1, 30:1, 50:1 and 100:1 were 91.7±1.9% (n = 3,389), 44.0±1.7% (n = 3,105), 25.3±3.4% (n = 2,902), 10.3±0.2% (n = 4,949), 7.6±0.7% (n = 4,042), 1.0±0.4% (n = 30,103) and 0.008±0.002% (n = 29,432), respectively. The OFR had a significant effect on fertility of the females (*F* = 85.6; d.f. = 1, 16; *P* < 10^−3^) (Fig 2). At the sterile:fertile male ratios of 30:1 and 50:1, egg hatch was reduced to 7.6±1.2% and 1.0±0.7%, respectively.

According to the Probit linear regression generated, the estimated ratio of sterile:fertile males to induce 50%, 90% and 99% sterility, with the 95% confidence intervals, were 1.6 (0.9; 2.9), 9.6 (6.7; 13.9), and 41.7 (27.1; 64.3), respectively.

### Sterility induction under field cage conditions

The results from the field cage tests are shown in Tables 6 and 7. The different OFRs, both when releasing sterile males and females jointly and when releasing males only, had a significant effect on the number of pupae recovered.

**Table 6:** Means (±SE) of number of pupae recovered, reduction in pupal production and pupae obtained per kg of *papaya* fruit infested by *Anastepha fraterculus* at different ratios of sterile: Fertile males with the presence of sterile females in field cages.

Ratio (sterile ♂: fertile ♂)	Number of pupae	Reduction in pupal yield (%)	Number of pupae/kg of fruit
**Control**	115.0 ± 19.1	-	233 ± 39.0
**1:1**	49.3 ± 1.9	57.1 ± 1.6	99.9 ± 4.0
**10:1**	23.8 ± 14.7	79.3 ± 12.8	48 ± 29.0
**42:1**	5.3 ± 3.3	95.4 ± 2.8	10.7 ± 6.6
**50:1**	2.4 ± 0.67	97.9 ± 0.5	4.8 ± 1.3
**100:1**	0.25 ± 0.2	99.8 ± 0.2	0.51 ± 0.5
**Regression Analysis**	*ln* y = 4.7–0.12 x r^2^ = 0.93 *F*_1,17_ = 200.2; *P* < 10^−3^	*ln* y = 4.6–75.2 *e*^-x^ r^2^ = 0.89 *F*_1,15_ = 123.9; *P* < 10^−3^	*ln* y = 5.4–0.12 x r^2^ = 0.92 *F*_1,17_ = 201.4; *P* < 10^−3^

**Table 7:** Means (±SE) of number of pupae recovered, reduction in pupal production and pupae obtained per kg of *papaya* fruit infested by *Anastepha fraterculus* at different ratios of sterile: Fertile males without sterile females in field cages.

Ratio (sterile ♂: fertile ♂)	Number of pupae	Reduction in pupal yield (%)	Number of pupae/kg of Fruit
**Control**	119.6 ± 15.3	-	242.4 ± 31.0
**1:1**	48.0 ± 15.9	59.9 ± 13.3	97.3 ± 32.2
**10:1**	19.0 ± 4.7	84.1 ± 4.0	38.5 ± 9.6
**42:1**	3.1 ± 1.8	97.4 ± 1.5	6.3 ± 3.8
**50:1**	1.33 ± 0.95	98.9 ± 0.8	2.7 ± 1.9
**100:1**	0.14 ± 0.1	99.9 ± 0.1	0.3 ± 0.2
**Regression Analysis**	*ln* y = 4.8–0.15 x r^2^ = 0.96 *F*_1,17_ = 350.7; *P* < 10^−3^	*ln* y = 4.6–62.9 *e*^-x^ r^2^ = 0.92 *F*_1,15_ = 160.6; *P* < 10^−3^	*ln* y = 5.5–0.15 x r^2^ = 0.96 *F*_1,17_ = 350.0; *P* < 10^−3^

The reduction in pupal yield was higher than 95% for the 42:1, 50:1 and 100:1 ratios, and it increased as the number of sterile males per cage increased. According to the regression equations obtained (S8 Fig), both ratios of 42:1 and 50:1 could induce a reduction in pupal yield of 99.5%. Very few larvae developed in the papaya fruits that were exposed in the cages with the 100:1 ratio (*i*.*e*. only 0.3–0.5 pupae.kg^-1^ of fruit were obtained), and they came probably from eggs laid by one or two females that mated with nonirradiated males.

The sterile females apparently did not distract the sterile males. The slopes from the curves for number of pupae (*t* = 0.73, d.f. = 33; P = 0.47) and number of pupae per kg of fruit (*t* = 0.85, d.f. = 33; *P* = 0.39) did not differ significantly in the presence or absence of sterile females (S7 and S9 Figs). Although not significant (*t* = 0.27, d.f. = 29; *P* = 0.79), the mean percentages of reduction in pupal production were slightly higher for all ratios in the absence of the sterile females (Table 7 and S8 Fig).

## Discussion

As the SIT relies upon inundative releases of sterile mass-reared insects, it follows that choosing the optimum sterilizing dose is one of the principal steps of the technique. When bisexual strains are used, the minimum dose chosen must lead to full sterility in females and sterility levels close to 100% in males, aiming to preserve the vigour of the later ones [6, 7]. In view of the radiation studies conducted in the last decade, this was the study that evaluated the largest range of radiation doses for *A*. *fraterculus*. Assurance that the batches of pupae received the desired target doses was achieved by measuring the optical density of the dosimeter films after each exposure [12].

The regression analyses of Probit transformed sterility against log radiation dose developed for both sexes here showed that the estimated SD_99_ is ca. 39 Gy for males and 23 Gy for females (Table 3). The SD_99_ calculated for males by Mastrangelo et al. [11] were practically the same (*i*.*e*. 36.3–37.8 Gy), whereas the SD_99_ for females were not (*i*.*e*. 57.3–57.8 Gy). The results from the sterilization tests and the measurements of irradiated ovaries (Tables 3 and 5) confirm, thus, that the sterilizing doses calculated for females by Mastrangelo et al. [11] were overestimated due to the use of few doses and smaller egg sample sizes at high doses to fit the regression lines.

In agreement with our results, Allinghi et al. [10] found that pupal age up to 96 h prior to adult emergence does not significantly affect male sterility. In the sterilization tests conducted by these authors, they observed that the fertility of females crossed with males irradiated with 40 Gy was 3.0±2.0% (mean±SD) and that 100% sterility was induced by a dose of 70 Gy. They also verified that females irradiated with 40 Gy were unable to lay eggs, independently of the male to which they mated.

In the field cage tests conducted by Allinghi et al. [30], the residual fertility was only 0.75% in males but 2.17% in females after exposure to 40 Gy. This 2% fertility obtained from crosses between irradiated laboratory females and fertile wild males (*i*.*e*. 1 egg hatched out of 46 eggs collected) contrasts with the results from this study, Allinghi et al. [10] and Mastrangelo et al. [11] and it might have occurred by the accidental release of an under-dosed female or another experimental error. Bartolucci et al. [31] showed that 70 Gy caused complete and irreversible ovary atrophy, but our results demonstrated that this effect could be provoked with doses equal to or higher than 25 Gy (Fig 1).

The values of emergence and fliers (Table 4) were equivalent or higher than the minimum post-irradiation percentages specified for *Anastrepha ludens* (Loew), *Anastrepha obliqua* (Macquart) and *Anastrepha suspensa* (Loew) by the FAO/IAEA/USDA [13], and sex ratio did not diverge from 1:1, irrespectively of the age at which pupae were exposed to radiation. The mating competitiveness indices (S4 to S6 Figs) indicated that males irradiated with 40 Gy and nonirradiated males were competing equally for the fertile females. Several studies with *A*. *fraterculus* have showed that irradiation at 40–70 Gy does not affect its mating competitiveness [11, 30, 32], survival [33], sexual maturation [34], latency to mate, sperm transfer and remating behavior [35] under laboratory or field cage conditions.

In view of the sterility data and the values of the quality control parameters obtained here, the radiation treatment for *A*. *fraterculus* should be reviewed. The majority of operational SIT programmes adopt radiation doses that induce 99.9% or 100% sterility. For this purpose, Allinghi et al. [10] recommended that 70 Gy should be applied to pupae 48 h before emergence and this treatment has been used for most studies since then [36]. However, lower doses can yield more effectiveness for SIT field operations [37]. We believe that a sterilizing dose lower than the currently applied should be recommended for SIT projects against *A*. *fraterculus*, with any residual fertility being more than compensated by the high quality of the released flies. The long distances between the sterilization facilities and release sites in Brazil lead us to propose a protocol which consists in irradiating pupae 72 h before emergence with 40 Gy of gamma or X-rays.

This protocol will be adopted by the MOSCASUL center during its field trials in southern Brazil, but the optimum dose of 40 Gy should not be recommended blindly for all of the cryptic species of the *A*. *fraterculus* complex. This study and most of the Argentinean ones have tested only the Brazilian-1 morphotype. The three Brazilian morphotypes, for example, are distinct from the Peruvian populations [19, 38–41]. Gonzalez et al. [42] determined a sterilizing dose of 38.9 Gy for males, but they recommended 58.4 Gy for both sexes of the Peruvian strain tested. Accurate and consistant radiation studies with local morphotypes, therefore, are advised.

Regarding the sterile release density for *A*. *fraterculus*, this is the first study that brings information on this matter. The sterility induction verified in laboratory and field cages increased with the increase in the sterile:fertile ratio. Egg sterility levels increased from 56% to 89.7% between the 1:1 and 10:1 ratios (Fig 2), with the Probit analysis indicating that a 42:1 ratio would be sufficient to induce 99% sterility. This level of sterility, however, was more easily reached only by the OFR of 50:1 in the field cages (Tables 6 and 7). Assuming that the results from our field cage tests could reasonable be extrapolated to open field conditions, target populations could be suppressed by 90% or more with OFRs starting at 30:1. Our data suggests that an optimum sterile:fertile ratio in a SIT field trial against *A*. *fraterculus* should be 50 sterile males per 1 wild fly.

Most of the studies that estimate OFRs have been performed for *C*. *capitata* and *Bactrocera* spp. [15]. Steiner [43] proposed 20:1 as the critical OFR for *Bactrocera dorsalis* (Hendel) and *Zeugodacus cucurbitae* (Coquillett), while Villasenor et al. [44] suggested 80:1 as the minimum OFR for controlling *C*. *capitata*. After comparing existing field data, Shelly & McInnis [15] reported that *Z*. *cucurbitae* populations were reduced more than 99% at OFRs between 50:1 and 100:1 in Japan, whereas *C*. *capitata* populations were suppressed by 50–93% at an OFR of 160:1 in Nicaragua and ratios between 100:1 and 400:1 in Hawaii.

Only two studies tried to determine the relationship between induction of sterility and OFR for *Anastrepha* species. Flores et al. [16] used mass-reared *A*. *ludens* flies in large field cages, finding 85% and 90% sterility at OFRs of 30:1 and 100:1, respectively. These authors indicated that the optimal OFR for *A*. *ludens* should be 30:1. Flores et al. [17] showed that a 10:1 sterile:wild ratio was sufficient to induce ca. 88% sterility in wild *A*. *obliqua* when releasing only males, and a similar result was verified at the ratio of 30:1 when releasing sterile males and females jointly.

Due to our specific experimental conditions, higher OFRs may prove to be required under open field conditions. Confining flies in the field cages could provide some advantage to the sterile males by removing their need to disperse long distances in the environment to locate the wild females. Nevertheless, the fly densities applied in our field cages (*i*.*e*. densities ranged from 3 to 313 flies/m^2^) should neither be considered high, as overcrowded conditions in rearing cages for the tested strain occur only at densities higher than 4,000 flies/m^2^ (Mastrangelo, unpublished data), nor resource limited (adult diet and water were offered *ad libitum*). The efficacy of the sterile males could have been overestimated in our cages due to the exclusive use of domesticated flies. Wild females can be more choosy before mating [38, 45, 46], but the field cage conditions could also have favored the remating of nonirradiated females with the few fertile males released [35, 47], which could help to explain the recovery of a few pupae even at the OFR of 100:1. Future experiments trying to optimize the OFR for *A*. *fraterculus* might benefit from the use of wild strains and marked flies.

Additionally, it was not possible to conclude in this study that male only releases would provoke a higher degree of induced sterility in *A*. *fraterculus*, whilst bisexual releases of other species tend to be far less efficient [15]. Orozco et al. [48] demonstrated that the presence of sterile females of *A*. *ludens* diverted the attention of the sterile males, negatively influenced the proportion of mating with wild females and led to lower levels of induced sterility. Flores et al. [16, 17], however, did not find significant differences between bisexual and male-only releases. New attempts to precisely link sterile male only releases with sterility induction under different OFRs should be encouraged. The use of a GSS recently developed by the IAEA based on pupal color dimorphism [49] may be useful to achieve this.

In conclusion, the data generated here have a great practical value for the decision-makers of SIT field trials, proving that the optimum dose of 40 Gy render females incapable of laying eggs and giving a preliminary idea about the minimum number of sterile males that must be released to start driving the target population downward to reproductive collapse. In addition, due to the fact that flies younger than 7 days cannot be easily distinguished on the basis of ovary development regardless of the radiation dose, the ovarian dissection technique [31] should be used with caution by those willing to confirm sterility of trapped females that are released in the field but not adequately marked with fluorescent dyes. Further investigations about the effects of hypoxia on the sterilizing dose, cold storage and shipment over long distances on the quality of the sterile flies, and large-field tests are still essential to the SIT’s effectiveness against *A*. *fraterculus*. As soon as these issues are resolved, SIT programmes for this fruit fly can come closer to becoming a reality in South America.

## Supporting information

S1 Fig Linear regression of Probit transformed sterility on log dose for males (in blue) and females (in red) of *Anastrepha fraterculus* whose pupae were irradiated at 72 h before adult emergence. (TIF)

S2 Fig Linear regression of Probit transformed sterility on log dose for males (in blue) and females (in red) of *Anastrepha fraterculus* whose pupae were irradiated at 48 h before adult emergence. (TIF)

S3 Fig Linear regression of Probit transformed sterility on log dose for males (in blue) and females (in red) of *Anastrepha fraterculus* whose pupae were irradiated at 24 h before adult emergence. (TIF)

S4 Fig Index of sexual isolation for irradiated *Anastrepha fraterculus*. (TIF)

S5 Fig Relative performance indices for irradiated *Anastrepha fraterculus*. (TIF)

S6 Fig Relative isolation index for irradiated *Anastrepha fraterculus*. (TIF)

S7 Fig Relationship between number of pupae collected and the sterile: Fertile male ratio under field cage conditions, using male-only and male and female releases. (TIF)

S8 Fig Relationship between reduction in pupal yield and the sterile: Fertile male ratio under field cage conditions, using male-only and male and female releases. (TIF)

S9 Fig Relationship between number of pupae obtained per kilogram of fruit and the sterile: Fertile male ratio under field cage conditions, using male-only and male and female releases. (TIF)

## References

[1] Dyck VA, Hendrichs J, Robinson AS (2005) Sterile insect technique: principles and practice in area-wide integrated pest management. Springer, Berlin, Germany.

[2] Hendrichs J, Vreysen MJB, Enkerlin WR, Cayol JP (2005) Strategic options in the use of the sterile insect technique In: Dyck VA, Hendrichs J, Robinson AS, editors. Sterile Insect Technique: principles and practice in area-wide integrated pest management. Pays-Bas: Springer; 2005. p. 563–600.

[3] Orozco-Davila D, Quintero L, Hernandez E, Solis E, Artiaga T, Hernandez R, et al (2017) Mass rearing and sterile insect releases for the control of *Anastrepha* spp. pests in Mexico—a review. Entomologia Experimentalis et Applicata 164: 176–187. 10.1111/eea.12581

[4] Kovaleski A, Mumford J (2007) Pulling out the Evil by the Root: the Codling Moth Cydia pomonella Eradication Programme in Brazil In: Vreysen MJB, Robinson AS, Hendrichs J, editors. Area-wide control of insect pests, from research to field implementation. Pays-Bas: Springer; 2007. p. 581–590.

[5] Costa MLZ, Pacheco MG, Lopes LA, Botteon VW, Mastrangelo T (2016) Irradiation of *Anastrepha fraterculus* (Diptera: Tephritidae) Eggs to Inhibit Fly Emergence in the Mass-Rearing of *Diachasmimorpha longicaudata* (Hymenoptera: Braconidae). Journal of Insect Science 16: 98–106. 10.1093/jisesa/iew071 27638956 PMC5026477

[6] Bakri A, Mehta K, Lance DR (2005) Sterilizing insects with ionizing radiation In: Dyck VA, Hendrichs J, Robinson AS, editors. Sterile Insect Technique: principles and practice in area-wide integrated pest management. Pays-Bas: Springer; 2005. p. 233–269.

[7] Robinson AS (2005) Genetic basis of the sterile insect technique In: Dyck VA, Hendrichs J, Robinson AS, editors. Sterile Insect Technique: principles and practice in area-wide integrated pest management. Pays-Bas: Springer; 2005. p. 95–114.

[8] Calkins CO, Parker AG (2005) Sterile insect quality In: Dyck VA, Hendrichs J, Robinson AS, editors. Sterile Insect Technique: principles and practice in area-wide integrated pest management. Pays-Bas: Springer; 2005. p. 269–296.

[9] [IDIDAS] International Database for Insect Disinfestation And Sterilization. 2017. International database for insect disinfestation and sterilization. International Database for Insect Disinfestation And Sterilization. (http://www.ididas.iaea.org/IDIDAS/default.htm).

[10] Allinghi A, Gramajo C, Willink E, Vilardi JC (2007) Induction of sterility in *Anastrepha fraterculus* (Diptera: Tephritidae) by gamma radiation. Florida Entomologist 90: 96–102. 10.1653/0015-4040(2007)90[96:IOSIAF]2.0.CO;2

[11] Mastrangelo T, Parker AG, Jessup A, Pereira R, Orozco-Dávilla D, Islam A, et al (2010) A new generation of X ray irradiators for insect sterilization. Journal of Economic Entomology 103: 85–94. 10.1603/EC09139 20214372

[12] [IAEA] International Atomic Energy Agency. (2004). Dosimetry system for SIT: standard operating procedure for Gafchromic film. International Atomic Energy Agency, Vienna, Austria. (http://www.naweb.iaea.org/nafa/ipc/public/ipc-gafchromic-dosimetry-sit.html).

[13] [FAO/IAEA/USDA] Food and Agriculture Organization of the United Nations/International Atomic Energy Agency/United States Department of Agriculture. 2014. Product Quality Control for Sterile Mass-Reared and Released Tephritid Fruit Flies, Version 6.0. International Atomic Energy Agency, Vienna, Austria. 164 pp.

[14] Knipling EF (1979) The basic principles of insect population suppression and management Agriculture Handbook, Number 512. Science and Education Administration, United States Department of Agriculture, Washington, D.C., USA.

[15] Shelly T, McInnis D (2016) Sterile Insect Technique and Control of Tephritid Fruit Flies: Do species with complex courtship require higher overflooding ratios? Annals of the Entomological Society of America 109: 1–11. 10.1093/aesa/sav101

[16] Flores S, Montoya P, Toledo J, Enkerlin W, Liedo P (2014) Estimation of Populations and Sterility Induction in *Anastrepha ludens* (Diptera: Tephritidae) Fruit Flies. Journal of Economic Entomology 107: 1502–1507. 10.1603/EC13398 25195442

[17] Flores S, Gómez‐Escobar E, Liedo P, Toledo J, Montoya P (2017). Density estimation and optimal sterile‐to‐wild ratio to induce sterility in *Anastrepha obliqua* populations. Entomologia Experimentalis et Applicata 164: 284–290. 10.1111/eea.12580

[18] Walder JMM, Morelli R, Costa KZ, Faggioni KM, Sanches PA, Paranhos BAJ, et al (2014) Large scale artificial rearing of Anastrepha sp.1 aff. fraterculus (Diptera: Tephritidae) in Brazil. Scientia Agricola 71: 281–286. 10.1590/0103-9016-2013-233

[19] Dias VS, Silva JC, Lima KM, Petitinga CSCD, Hernández-Ortiz V, Laumann RA, et al (2016) An integrative multidisciplinary approach to understanding cryptic divergence in Brazilian species of the *Anastrepha fraterculus* complex (Diptera: Tephritidae). Biological Journal of the Linnean Society 117: 725–746. 10.1111/bij.12712

[20] Mehta K, Parker A (2011). Characterization and dosimetry of a practical X-ray alternative to self-shielded gamma irradiators. Radiation Physics and Chemistry 80: 107–113.

[21] Nation JL (1974) The structure and development of two sex specific glands in male Caribbean fruit flies. Annals of the Entomological Society of America 67(5): 731–734. 10.1093/aesa/67.5.731

[22] Bron IU, Jacomino AP (2006) Ripening and quality of 'Golden' papaya fruit harvested at different maturity stages. Brazilian Journal of Plant Physiology 18: 389–396. 10.1590/S1677-04202006000300005

[23] Machota JR, Bortoli LC, Tolotti A, Botton M (2010) Técnica de criação de *Anastrepha fraterculus* (Wied., 1830) (Diptera: Tephritidae) em laboratório utilizando hospedeiro natural. Bento Gonçalves: Boletim de Pesquisa e Desenvolvimento, Embrapa Uva e Vinho.

[24] Abbott WS (1925) A method of computing the effectiveness of an insecticide. Journal of Economic Entomology 18: 265–267.

[25] Sokal RR, Rohlf FJ (1995) Biometry: The principles and practice of statistics in biological research, 3^rd^ edition W. H.Freeman & Co., New York.

[26] Rhode RH, Simon J, Perdomo A, Gutierrez J, Dowling CF Jr, Lindquist DA (1971) Application of the sterile-insect-release technique in Mediterranean fruit fly suppression. Journal of Economic Entomology 64: 708–713. 10.1093/jee/64.3.708

[27] Bartlett MS (1937) Properties of sufficiency and statistical tests. Proceedings of the Royal Statistical Society Series A 160: 268–282.

[28] Shapiro SS, Wilk MB (1965) An analysis of variance test for normality (complete samples). Biometrika 52: 591–611.

[29] SAS Institute Inc. (2013) SAS Software Version 9.4. Cary, NC.

[30] Allinghi A, Calcagno G, Petit-Marty N, Gomez-Cendra PV, Segura DF, Vera MT, et al (2007) Compatibility and competitiveness of a laboratory strain of *Anastrepha fraterculus* (Diptera: Tephritidae) after irradiation treatment. Florida Entomologist 90: 27–32. 10.1653/0015-4040(2007)90[27:CACOAL]2.0.CO;2

[31] Bartolucci AF, Vera MT, Yusef V, Oviedo A (2006) Morphological characterization of the reproductive system of irradiated Anastrepha fraterculus. In Proc. 7th International Symposium of Fruit Flies of Economic Importance, 10–15 September 2006, Salvador, Brazil. Biofábrica Moscamed Brasil; Sugayama, R.L., Zucchi, R.A., Ovruski, S.M., Sivinski, J., p. 45–52.

[32] Liendo MC, Devescovi F, Bachmann GE, Utgés ME, Abraham S, Vera MT, et al (2013) Precocious sexual signalling and mating in *Anastrepha fraterculus* (Diptera: Tephritidae) sterile males achieved through juvenile hormone treatment and protein supplements. Bulletin of Entomological Research 103: 1–13. 10.1017/S0007485312000442 22929968

[33] Gomez-Cendra PV, Segura DF, Allinghi A, Cladera JL, Vilardi JC (2007) Comparison of longevity between a laboratory strain and a natural population of *Anastrepha fraterculus* (Diptera: Tephritidae) under field cage conditions. Florida Entomologist 90: 147–153. 10.1653/0015-4040(2007)90[147:COLBAL]2.0.CO;2

[34] Segura DF, Utgés ME, Liendo MC, Rodríguez MF, Devescovi F, Vera MT, et al (2013) Methoprene treatment reduces the pre-copulatory period in *Anastrepha fraterculus* (Diptera: Tephritidae) sterile males. Journal of Applied Entomology 137: 19–29. 10.1111/j.1439-0418.2010.01534.x

[35] Abraham S, Liendo MC, Devescovi F, Peralta PA, Yusef V, Ruiz J, et al (2013) Remating behavior in *Anastrepha fraterculus* (Diptera: Tephritidae) females is affected by male juvenile hormone analog treatment but not by male sterilization. Bulletin of Entomological Research 103: 310–317. 10.1017/S0007485312000727 23340454

[36] Alba MG, Segura D, Terrada MM, Lopez S (2016) Estudio Comparativo Sobre el efecto de la Radiación X y Gamma sobre pupas de Anastrepha fraterculus (Wied). In D. Quiroga (ed.), Libro de Resumenes: 9th Meeting of Tephritid Workers of the Western Hemisphere, 17–21 October 2016, Buenos Aires, Argentina.

[37] Parker A, Mehta K (2007) Sterile insect technique: a model for dose optimization for improved sterile insect quality. Florida Entomologist 90: 88–95. 10.1653/0015-4040(2007)90[88:SITAMF]2.0.CO;2

[38] Vera MT, Caceres C, Wornoayporn V, Islam A, Robinson AS, De La Vega MH, et al (2006) Mating incompatibility among populations of the South American fruit fly *Anastrepha fraterculus* (Diptera: Tephritidae). Annals of the Entomological Society of America 99: 387–397. 10.1603/0013-8746(2006)099[0387:MIAPOT]2.0.CO;2

[39] Rull J, Abraham S, Kovaleski A, Segura DF, Mendoza M, Clara Liendo M, et al (2013) Evolution of pre-zygotic and post-zygotic barriers to gene flow among three cryptic species within the *Anastrepha fraterculus* complex. Entomologia Experimentalis et Applicata 148: 213–222. 10.1111/eea.12094

[40] Devescovi F, Abraham S, Roriz AKP, Nolazco N, Castaneda R, Tadeo E, et al (2014) Ongoing speciation within the *Anastrepha fraterculus* cryptic species complex: the case of the Andean morphotype. Entomologia Experimentalis et Applicata 152: 238–247. 10.1111/eea.12219

[41] Hernández-Ortiz V, Canal NA, Tigrero-Salas JO, Ruíz-Hurtado FM, Dzul-Cauich JF (2015) Taxonomy and phenotypic relationships of the *Anastrepha fraterculus* complex in the Mesoamerican and Pacific Neotropical dominions (Diptera, Tephritidae). In De Meyer M., Clarke A.R., Vera M.T., Hendrichs J. (Eds.), *Resolution of Cryptic Species Complexes of Tephritid Pests to Enhance SIT Application and Facilitate International Trade*. ZooKeys, 540, 95 10.3897/zookeys.540.6027 26798256 PMC4714066

[42] Gonzalez J, Vargas C, Jara B (1971) Irradiación de *Anastrepha fraterculus* (Wied.). Revista Peruana de Entomologia 14: 77–79.

[43] Steiner LF (1969) A method of estimating the size of native populations of oriental, melon, and Mediterranean fruit flies, to establish the overflooding ratios required for sterile-male releases. Journal of Economic Entomology 62: 4–7. 10.1093/jee/62.1.4

[44] Villasenor A, Carrillo J, Zavala J, Stewart J, Lira C, Reyes J (2000) Current progress in the medfly program Mexico-Guatemala In: Tan KH, editor. Area-wide control of fruit flies and other insect pests. Penerbit Universiti Sains Malaysia, Pulau Pinang, Malaysia, p. 361–368.

[45] Gomez-Cendra PV, Calcagno G, Belluscio L, Vilardi JC (2011) Male courtship behavior of the South American fruit fly, *Anastrepha fraterculus*, from an Argentinean laboratory strain. Journal of Insect Science 11 (175): 1–18. 10.1673/031.011.17501 22958000 PMC3469206

[46] Abraham S, Goane L, Rull J, Cladera JL, Willink E, Vera MT (2011) Multiple mating in *Anastrepha fraterculus* females and its relationship with fecundity and fertility. Entomologia Experimentalis et Applicata 141: 15–24. 10.1111/j.1570-7458.2011.01160.x

[47] Vera MT, Wood R, Cladera JL, Gilburn A (2002) Remating frequency in the Mediterranean fruit fly (Diptera: Tephritidae) under laboratory conditions. Florida Entomologist 85: 156–164.

[48] Orozco D, Hernandez MR, Meza JS, Quintero JL (2013) Do sterile females affect the sexual performance of sterile males of *Anastrepha ludens* (Diptera: Tephritidae)? Journal of Applied Entomology 137: 321–326. 10.1111/j.1439-0418.2012.01748.x

[49] Meza JS, Caravantes S, Caceres C (2016) Development of a genetic sexing system for the South American fruit fly, Anastrepha fraterculus (Diptera: Tephritidae). In D. Quiroga (Ed.), Libro de Resumenes: 9th Meeting of Tephritid Workers of the Western Hemisphere, 17–21 October 2016, Buenos Aires, Argentina.

