# Supplementary figures and images for: Optimization of the sterilizing doses and overflooding ratios for the South American fruit fly
Source: PLoS One. 2018 Jul 20;13(7):e0201026. doi: 10.1371/journal.pone.0201026 (PMC6054417; doi:10.1371/journal.pone.0201026)

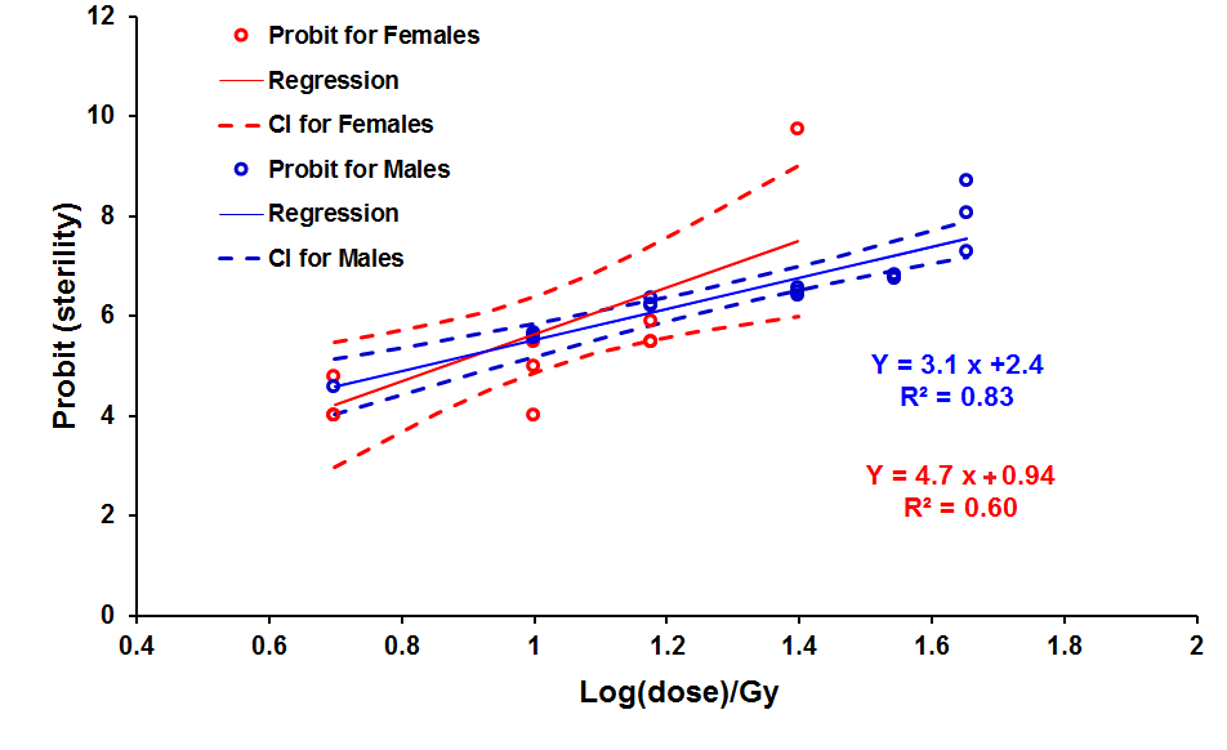

Supplement: S1 Fig — (TIF) [file pone.0201026.s001.tif]

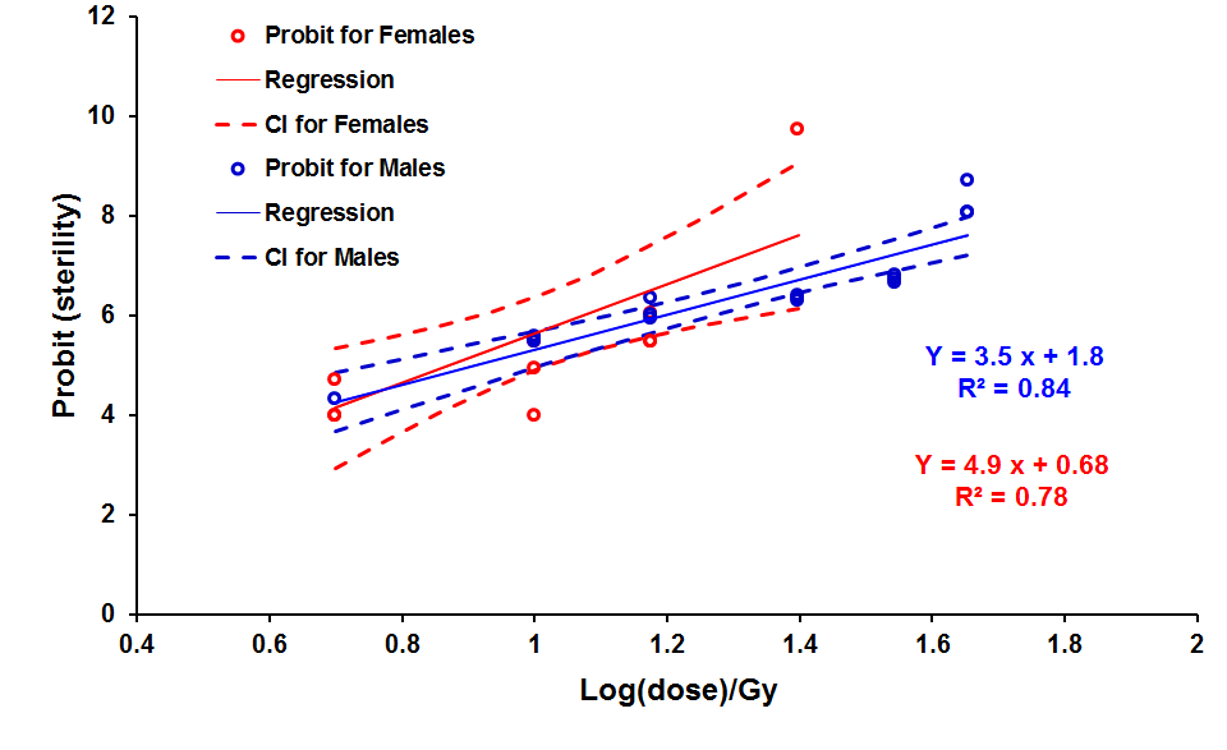

Supplement: S2 Fig — (TIF) [file pone.0201026.s002.tif]

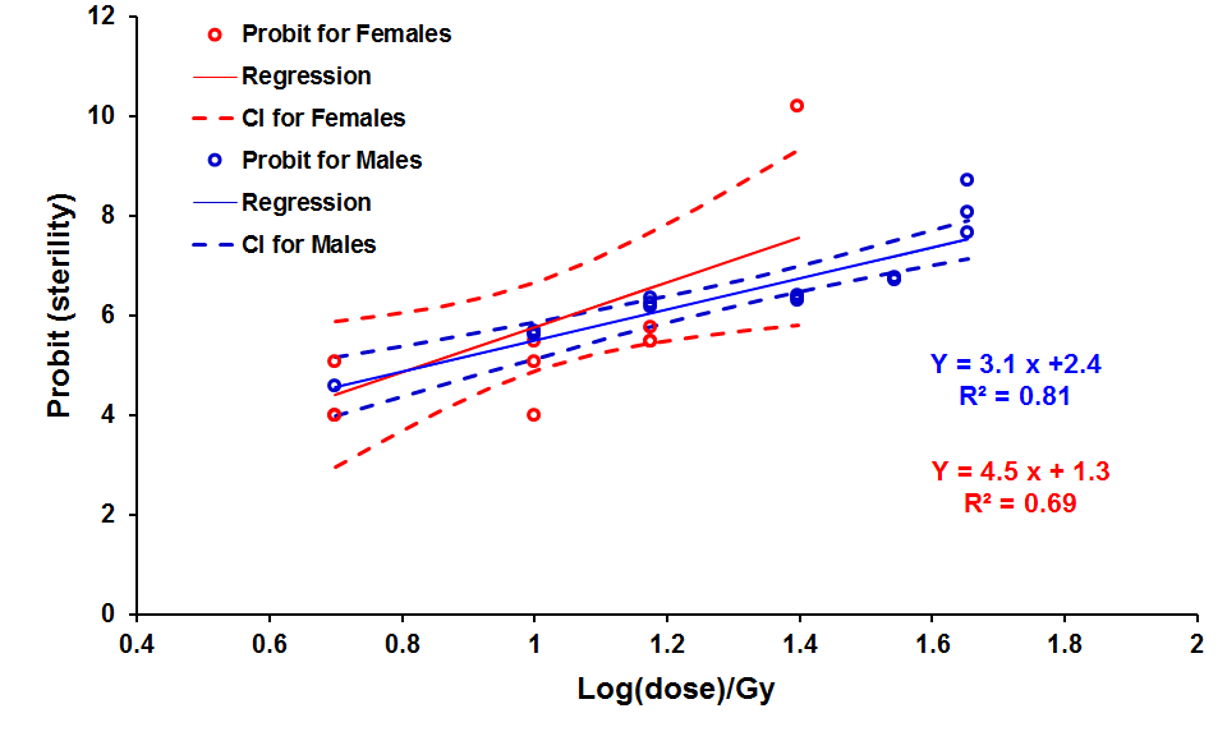

Supplement: S3 Fig — (TIF) [file pone.0201026.s003.tif]

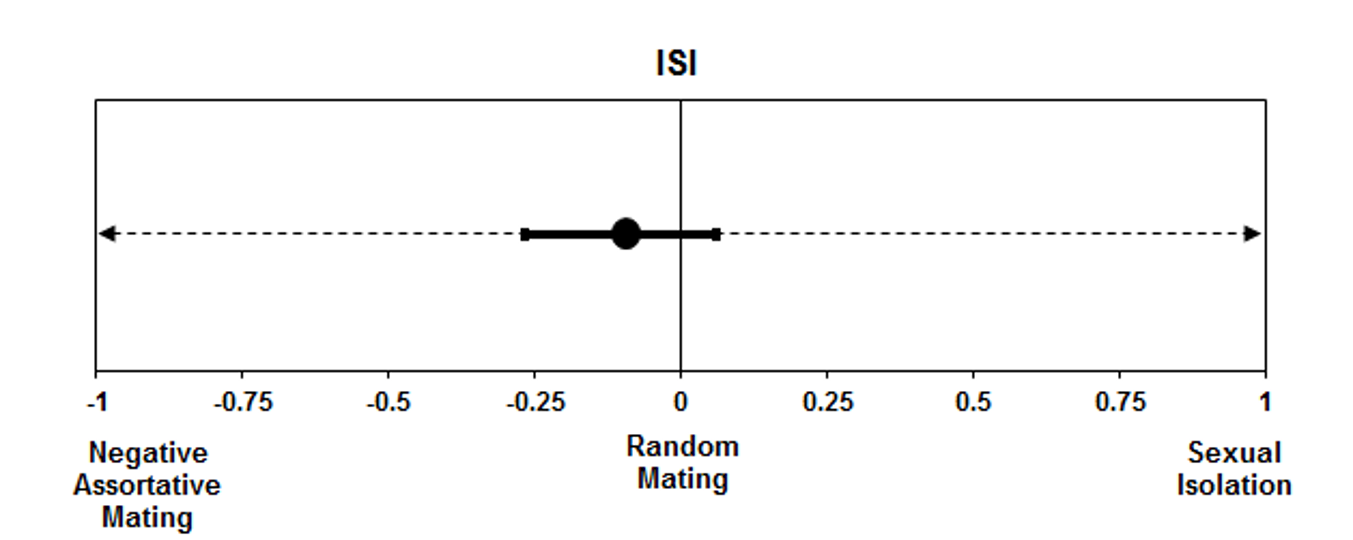

Supplement: S4 Fig — (TIF) [file pone.0201026.s004.tif]

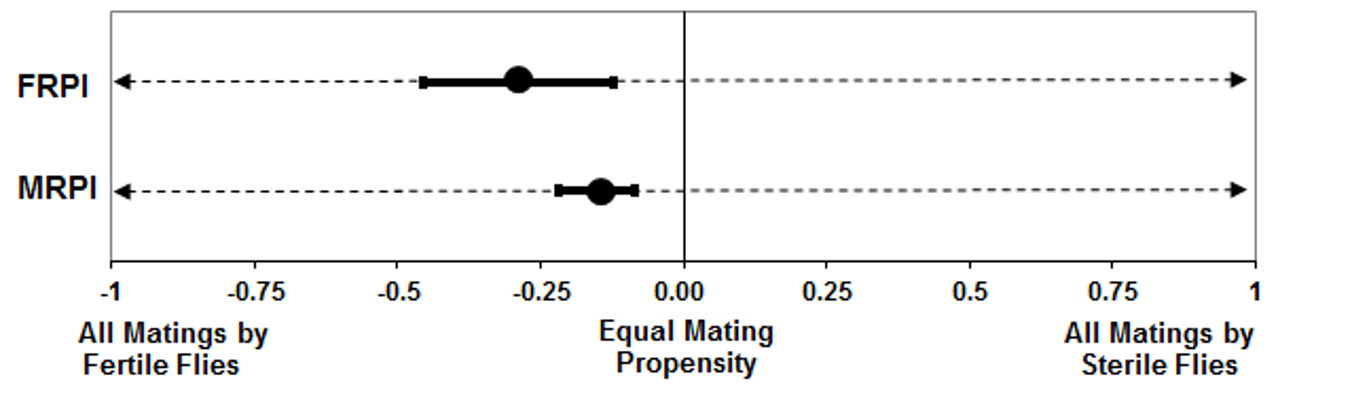

Supplement: S5 Fig — (TIF) [file pone.0201026.s005.tif]

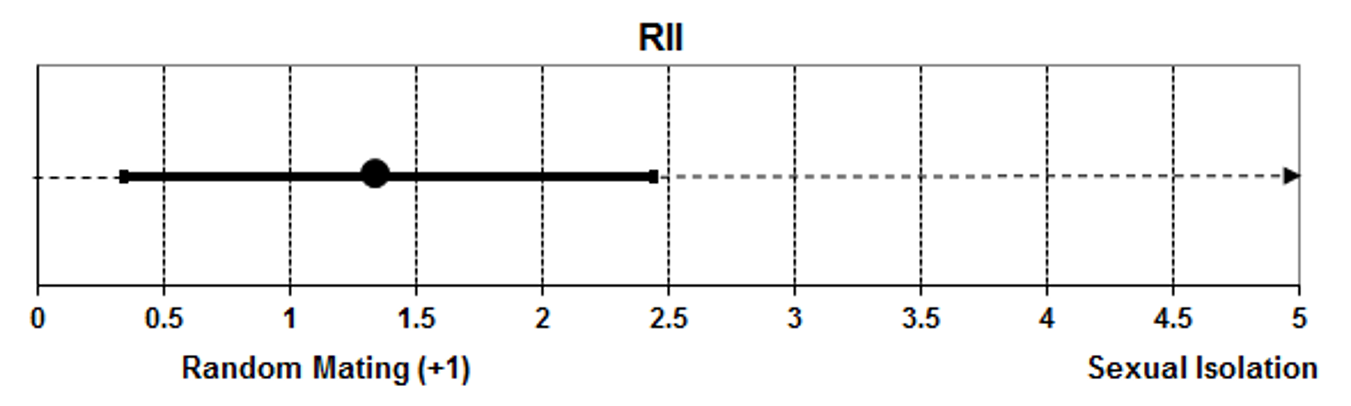

Supplement: S6 Fig — (TIF) [file pone.0201026.s006.tif]

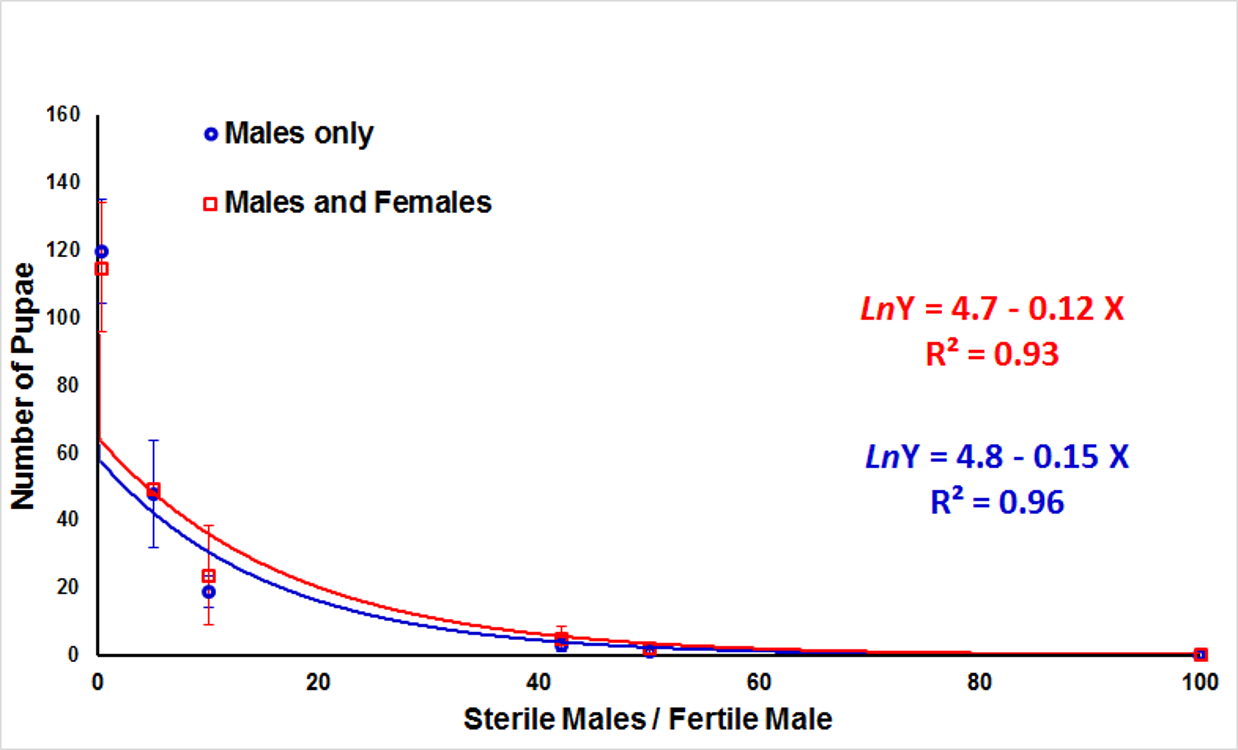

Supplement: S7 Fig — (TIF) [file pone.0201026.s007.tif]

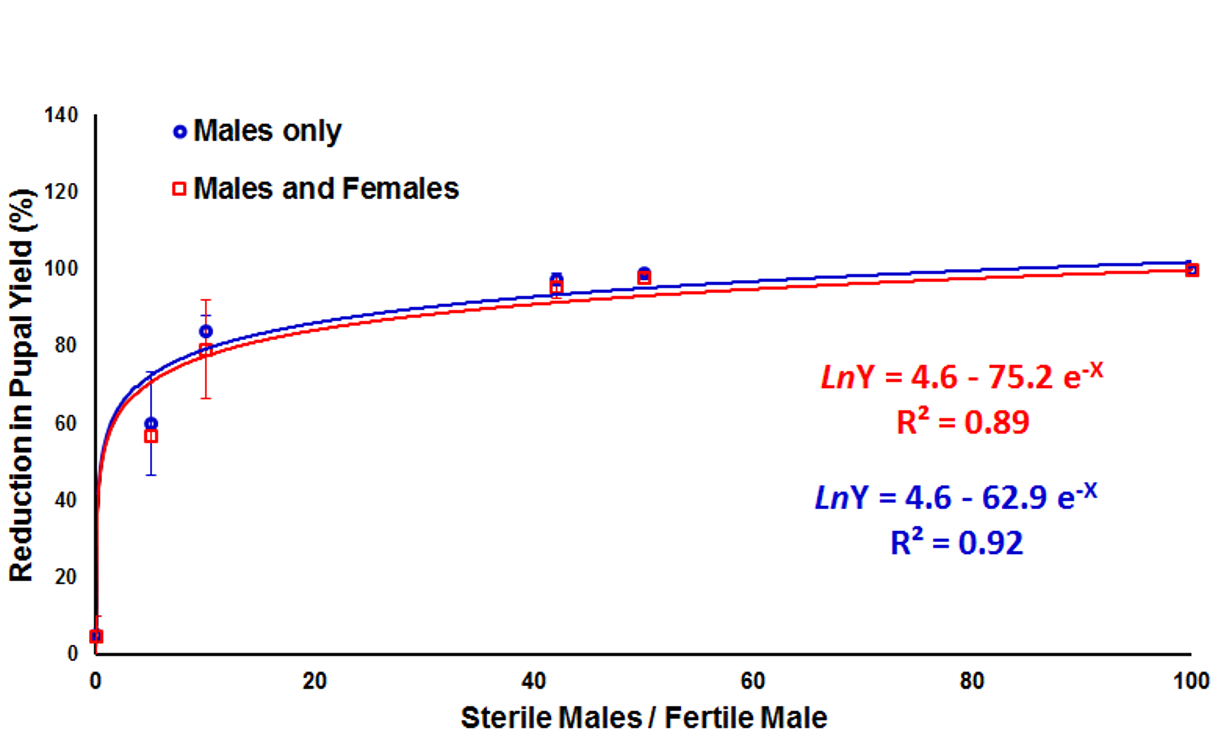

Supplement: S8 Fig — (TIF) [file pone.0201026.s008.tif]

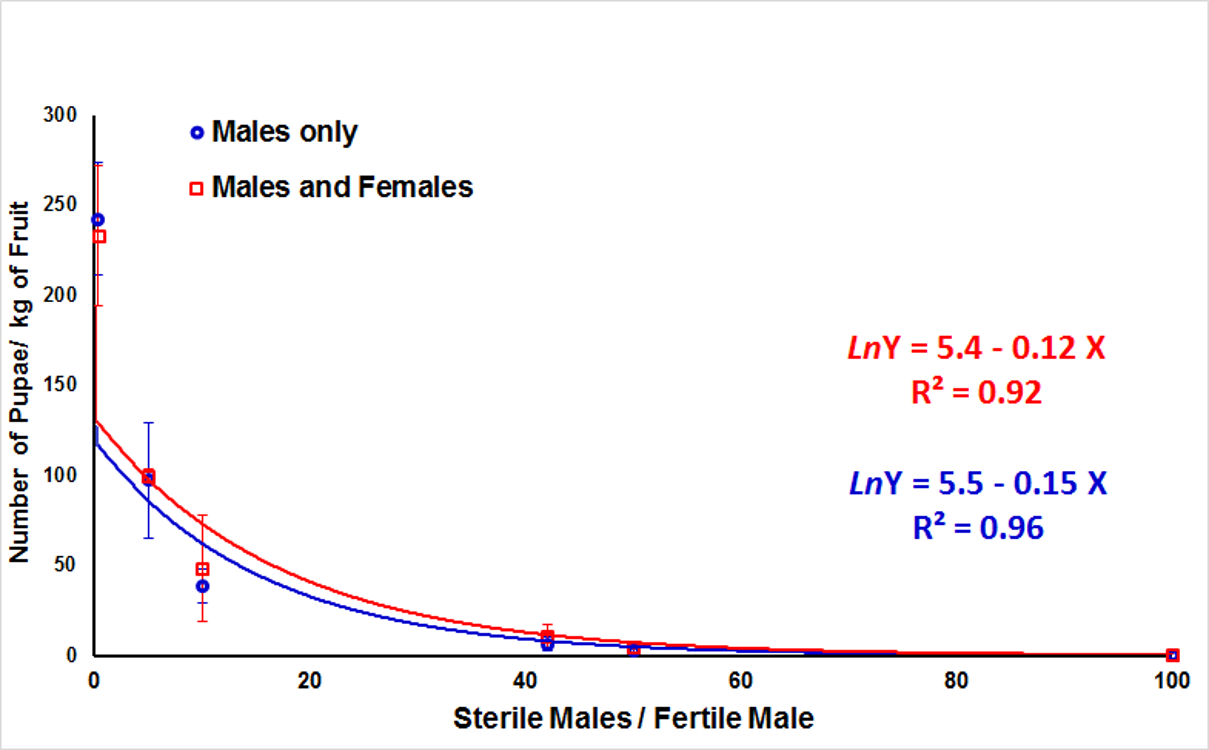

Supplement: S9 Fig — (TIF) [file pone.0201026.s009.tif]
